# The Role of STAT3 in Thyroid Cancer

**DOI:** 10.3390/cancers6010526

**Published:** 2014-03-06

**Authors:** Nadiya Sosonkina, Dmytro Starenki, Jong-In Park

**Affiliations:** Department of Biochemistry, Medical College of Wisconsin, 8701 Watertown Plank Road, Milwaukee, WI 53226, USA; E-Mails: nsosonkina@mcw.edu (N.S.); dstarenki@mcw.edu (D.S.)

**Keywords:** thyroid cancer, JAK, STAT3, interleukin 6, leukemia inhibitory factor

## Abstract

Thyroid cancer is the most common endocrine malignancy and its global incidence rates are rapidly increasing. Although the mortality of thyroid cancer is relatively low, its rate of recurrence or persistence is relatively high, contributing to incurability and morbidity of the disease. Thyroid cancer is mainly treated by surgery and radioiodine remnant ablation, which is effective only for non-metastasized primary tumors. Therefore, better understanding of the molecular targets available in this tumor is necessary. Similarly to many other tumor types, oncogenic molecular alterations in thyroid epithelium include aberrant signal transduction of the mitogen-activated protein kinase, phosphatidylinositol 3-kinase/AKT (also known as protein kinase B), NF-кB, and WNT/β-catenin pathways. However, the role of the Janus kinase (JAK)/signal transducer and activator of transcription (STAT3) pathway, a well-known mediator of tumorigenesis in different tumor types, is relatively less understood in thyroid cancer. Intriguingly, recent studies have demonstrated that, in thyroid cancer, the JAK/STAT3 pathway may function in the context of tumor suppression rather than promoting tumorigenesis. In this review, we provide an update of STAT3 function in thyroid cancer and discuss some of the evidences that support this hypothesis.

## 1. Introduction

Thyroid cancer is the most common neoplasm of the endocrine system, which originates from follicular thyrocytes or parafollicular C-cells of the thyroid gland (reviewed in [1]). Although relatively rare, thyroid cancer is the seventh most frequent human malignancy, and is increasing in incidence more rapidly than any other cancers. From 1995 to 2008, thyroid cancer age-adjusted incidence rates more than doubled in the U.S., whereas the incidence of most major cancers (lung, prostate, breast and colorectal) decreased during the same period [2]. Therefore, thyroid cancer is a growing health problem. Thyroid cancer is mainly treated by surgery and radioiodine remnant ablation, which is effective only for non-metastasized primary tumors. Metastatic and relapsed tumors are mostly incurable, requiring more advanced therapeutic modalities for patient survival.

Depending upon the cell origin and histological characteristics, thyroid carcinomas are generally classified to papillary thyroid carcinoma (PTC), follicular thyroid carcinoma (FTC), anaplastic thyroid carcinoma (ATC, poorly differentiated), and medullary thyroid cancer (MTC). As summarized in Table 1, most of these thyroid tumors arise from the follicular thyrocytes whereas MTC is the only C-cell-originated tumor, constituting the minor fraction of thyroid malignancies. While various alterations at molecular levels underlie the onset of thyroid carcinogenesis, activation of the mitogen-activated protein kinase (MAPK) and phosphatidylinositol 3-kinase (PI3K)/AKT pathways, often in close connection and cooperation, constitute the major mechanisms for the development and progression of most thyroid tumors (Table 1). Nevertheless, activation of these pathways is attributed to different genetic or epigenetic alteration of oncogenes and tumor suppressors. In about 65% to 70% of the follicular thyrocyte-derived tumors, mutated B-Raf, Ras, phosphatase and tensin homolog (PTEN), or PIK3CA drives the activation of the MAPK and PI3K pathways [3,4,5]. Ras protein activator like 1 (RASAL1) is a major tumor suppressor in thyroid cancer and its alterations can affect the MAPK pathway activity [6]. Moreover, oncogenically mutated forms of *RE*arranged during *T*ransfection (RET) receptor tyrosine kinase are also often detected in certain thyroid tumors and are supposed to drive the pathways. For example, various mutations in the cell surface receptor domain or the cytoplasmic kinase domain constitutively activate RET in about 95% of hereditary MTC and about 50% of sporadic MTC cases (reviewed in [7,8]). In about 20% of PTC patients, the kinase domain of RET is often fused to various unrelated genes by chromosomal rearrangements (known as RET/PTC) and becomes constitutively active (reviewed in [9]). In addition, activation of other receptor tyrosine kinases including epidermal growth factor receptor (EGFR), vascular endothelial growth factor receptor (VEGFR), and c-MET/hepatocyte growth factor receptor are also detected in a subset of MTC patients [10]. Despite the plethora of information on different signal transduction pathways in thyroid cancer, the information on the Janus kinase (JAK)/signal transducer and activator of transcription (STAT3) pathway is relatively limited. Intriguingly, emerging evidences suggest that the JAK/STAT3 pathway may have tumor suppressive effects on thyroid cancers, unlike its effects on many other cancer types.

**Table 1:** Molecular alterations detected in different thyroid tumor types *.

Tumor type	Cells of origin	Prevalence (% of total thyroid cancers)	Frequently detected genetic/epigenetic alterations	Frequently detected aberrant pathway signaling
Papillary thyroid carcinoma (PTC)	Follicular thyrocytes	80%–85%	*BRAF*^V600E^ (45%)	MAPK pathway PI3K/AKT pathway IDH1-associated metabolic pathways
			*RET/PTC* translocation (20%)	
			*IDH1* mutation (10%)	
			*EGFR* mutation (5%)	
			*RASAL1* mutation or hypermethylation (3%)	
			*PTEN* mutation (1%–2%)	
			*PIK3CA* mutations (1%–2%)	
Follicular thyroid carcinoma (FTC)	Follicular thyrocytes	10%–15%	*PAX8/PPARγ* rearrangement (40%–60%)	MAPK pathway PI3K/AKT pathway IDH1-associated metabolic pathways
			*HRAS, KRAS,* or *NRAS* mutation (30%–45%)	
			*RASAL1* mutation or hypermethylation (32%)	
			*PTEN* deletion (30%)	
			*PTEN* mutation (10%–15%)	
			*PIK3CA* mutation (5%–15%)	
			*IDH1* mutation (5%–15%)	
Poorly differentiated thyroid carcinoma (PDTC)	Follicular thyrocytes	5%–10%	*HRAS, KRAS,* or *NRAS* mutation (20%–40%)	MAPK pathway PI3K/AKT pathway WNT/β-catenin pathway p53-regulated pathways
			*CTNNB1* mutation (25%)	
			*TP53* mutation (25%)	
Anaplastic thyroid carcinoma (ATC)	Follicular thyrocytes	2%–3%	*BRAF*^V600E^ mutation (25%–50%)	MAPK pathway PI3K/AKT pathway WNT/β-catenin pathway p53-regulated pathways IDH1- associated metabolic pathways
			*HRAS, KRAS,* or *NRAS* (20%–30%)	
			*PTEN* mutation (10%–20%)	
			*PIK3CA* mutation (15%–25%)	
			*CTNNB1* mutation (60%–65%)	
			*TP53* mutation (70%–80%)	
			*IDH1* mutation (5%–15%)	
			*ALK* mutation (10%)	
			*RASAL1* mutations or hypermethylation (33%)	
Medullary thyroid cancer (MTC)	Parafollicular C-cells	2%–6%	*RET* mutation (99% of the familial, 30%–50% of the sporadic cases)	MAPK pathway
			*RAS* mutation (10% of the sporadic cases)	PI3K/AKT pathway

* This table was generated based on the reviews [1,10,11] and research articles [6,12,13,14,15,16]. For further details, refer to the cited reports. Abbreviations used are ALK, anaplastic lymphoma kinase; CTNNB1, cadherin-associated protein β1; EGFR; epithelial growth factor receptor; IDH, isocitrate dehydrogenase; MAPK, mitogen-activated protein kinase; PAX8, paired box 8; PI3K, phosphatidylinositol 3-kinase; PIK3CA, phosphatidylinositol-4,5-bisphosphate 3-kinase, catalytic subunit α; PPARγ, peroxisome proliferator-activated receptor-gamma; PTEN, phosphatase and tensin homolog; RASAL1, Ras protein activator like 1; RET, *RE*arranged in *T*ransformation; RET/PTC, RET/papillary thyroid carcinoma.

## 2. The JAK/STAT Pathway

The JAK/STAT pathway mediates signal transduction induced by cytokine receptors. Upon cytokine binding to the receptors, the receptor-associated cytoplasmic tyrosine kinase JAK cross-phosphorylates each other on tyrosine for activation. Active JAKs phosphorylate receptors to generate the docking site for the latent cytoplasmic factors, STATs (reviewed in [17]). STATs are then phosphorylated by JAKs, dissociate from the receptor, dimerize, and translocate into the nucleus to induce expression of a variety of genes, so called “cytokine-responsive genes” [18,19]. A number of hematopoietin subfamily of cytokines can bind to the cytokine receptors and activate the JAK/STAT pathway. To mediate this cytokine receptor signaling, human genome encodes four known JAKs and at least six STATs (reviewed in [19,20]).

### 2.1. STAT3

STAT3 is a latent gene regulatory protein, serving as a key effector of cytokine receptor/JAK/STAT signaling. STAT3 was originally discovered, along with STAT1 and STAT2, in a protein complex responsible for the regulation of interferon-dependent transcription [21,22]. STAT3 was also discovered as a transcription factor that binds to Interleukin (IL)-6-responsive elements located in the promoter region of various acute-phase genes [23], and then it was shown to be phosphorylated on its Tyr705 in response to IL-6 [24]; the history of early discovery of STATs has been recently reviewed in detail elsewhere [25]. STAT3 is constitutively expressed in a wide range of tissues. Nonetheless, immunohistochemical analysis of phosphorylated STAT3 on Tyr705 revealed that its nuclear presence is not ubiquitous but is selectively detected in three major cell types in human tissues, suggesting its tissue-specific function [26]. These cell types are (i) lymphoid and accessary cells in immune, digestive, and respiratory systems; (ii) glandular, mucosal, and secretory epithelium in digestive system, endocrine and reproductive systems, and endothelium of circulatory system (heart, veins, and capillaries); (iii) proliferative and reabsorption-active cells in productive, urinary, and integumentary systems [26]. Unlike other STATs, STAT3 deletion in mice results in lethality between 6.5 and 7.5 days of early embryogenesis, indicating that STAT3 may have more fundamental role than other STATs [27,28,29]. Indeed, while regulating a number of genes involved in the inflammatory response, STAT3 can also regulate different genes involved in apoptosis, differentiation, and stem cell maintenance, which indicates its wider involvement in the maintenance of cellular homeostasis (reviewed in [30]). 

### 2.2. Activation/Inactivation of STAT3

Phosphorylation of STAT3 on Tyr705 is the major mechanism of STAT3 activation, which is mediated by JAK upon stimulation of the heterodimeric gp130/cytokine-specific receptor complex by the IL-6 family of cytokines, including IL-6, leukemia inhibitory factor (LIF), ciliary neurotrophic factor, oncostatin M, IL-11, and cardiotrophin-1 (reviewed in [31]). STAT3 activation can also be mediated in a cytokine receptor-independent manner [17,20,32]. For example, activation of the receptor tyrosine kinases by epidermal growth factor or platelet-derived growth factor as well as expression of the oncogenic c-Src kinase or the small GTPase Ras can also induce STAT3 activation (additional signals that activate STAT3 are reviewed in [33]). Moreover, STAT3 phosphorylation on Tyr705 can also be mediated in a JAK-independent manner. For example, a recent work has demonstrated that the homodimer form of pyruvate kinase M2 can localize into the nucleus and phosphorylate STAT3 on Tyr705, which was necessary to upregulate MEK5 transcription and increase cell proliferation under a metabolic stress condition [34]. In addition to Tyr705 phosphorylation, Ser727 phosphorylation also has a role for STAT3 activity, particularly for maximizing the transcriptional activity of STAT3 [35,36]. Ser727 phosphorylation of STAT3 is mainly mediated by MAPK1/3 (also known as ERK1/2) in response to different growth factor signals, which often activate receptor tyrosine kinases. Therefore, STAT3 provides a converging point for the signal transduction mediated by cytokine receptors and receptor tyrosine kinases. Intriguingly, although phosphorylation of these two amino acid residues is expected to be synergistic for maximal STAT3 activation, it has been reported that Ser727 phosphorylation can prevent Tyr705 phosphorylation or even induces its dephosphorylation, suggesting the presence of “cross-regulation” to balance STAT3 activity between different pathways that activate STAT3 [35].

The activity of STAT3 should be precisely controlled for the maintenance of cellular homeostasis during development and in adults, which requires the participation of diverse negative regulators (reviewed in [32]). These negative regulators include (i) cytoplasmic tyrosine phosphatases, e.g., protein tyrosine phosphatase 1B, that dephosphorylate STAT proteins [37]; (ii) suppressors of cytokine signaling (SOCS) proteins that block the cytokine receptor [38]; (iii) proteins that inhibit activated STATs (PIAS) that interacts with tyrosine phosphorylated STATs and blocks their DNA binding [39]; (iv) naturally occurring truncated STAT proteins that can act as dominant-negative inhibitors by occupying DNA as non-functional transcription factor or by dimerizing with a wild-type STAT [40]. Loss of these negative regulators, particularly PIAS3 and SOCS, are known to contribute to abnormal STAT3 activation in leukemia, lymphoma, hepatocellular carcinoma and non-small cell lung carcinoma [41]. A study also demonstrated that SOCS-3 is frequently silenced by hypermethylation in human cancers [42].

### 2.3. Non-Canonical Activity of STAT3

Serving as a transcription factor upon activation via Tyr705 phosphorylation has been recognized as the canonical STAT3 function. However, STAT3 can also regulate transcription independently of Tyr705 phosphorylation. For example, similarly to the observation that unphosphorylated STAT1 could mediate LMP2 transcription in collaboration with IRF1 [43], unphosphorylated STAT3 can also regulate gene transcription in a complex with NF-κB [44,45,46,47]. In the STAT3/NF-κB complex, NF-κB provides DNA-binding and transactivation domains while STAT3 enables nuclear translocation of the complex [46]. Moreover, STAT3 is also subject to other posttranslational modifications in addition to phosphorylation and can mediate important cellular physiology independently of its transcriptional activating functions [25]. For example, non-tyrosine-phosphorylated and cytoplasmic-localized STAT3 could potentiate microtubule polymerization and cell movement by disrupting the interaction between microtubules and one of its partners, stathmin [48]. Recent studies have also demonstrated that STAT3 can localize into the mitochondria, where it controls the activity of the electron transport chain [49,50]. This STAT3 function in the mitochondria was shown to be important for Ras-induced transformation of mouse embryo fibroblasts [50]. These functions of STAT3 in respiration and Ras transformation required Ser727 phosphorylation but were independent of Tyr705 phosphorylation [50].

## 3. JAK/STAT Signaling in Different Cancer Types

STAT3 was originally identified as an oncogenic protein [51,52]. It has been shown that upregulated expression of STAT3 is associated with more malignant behavior of tumor cells and worse prognosis in a variety of human malignancies, e.g., cancers of the breast, prostate, ovary, lung, head and neck, esophagus, and brain [53,54,55,56,57,58,59,60,61]. In pancreatic cancer, STAT3 was shown to regulate angiogenesis and metastasis [62] and to promote cellular proliferation by accelerating G1/S-phase progression [63]. In gastric cancer, activation of STAT3 via the EGFR signaling pathway may contribute to tumor progression by increasing lymph node metastasis of the tumor [64]. Genetic/epigenetic alterations underlie aberrant STAT3 signaling in cancer and perturbation of positive or negative regulatory components in the JAK/STAT pathway that can cause a persistent STAT3 activation are often detected in different tumors (reviewed in [41]). Recent genome sequencing analyses have indeed revealed somatic mutations of the JAK/STAT pathway that occur in different tumor types [65,66,67]. Nonetheless, these mutations have not been reported yet in thyroid cancer.

STAT3 regulates many genes encoding cytokines, growth factors, and angiogenic factors, which in turn can activate STAT3 through their associated receptors. Therefore, persistent STAT3 activation in many cancers can establish a feed-forward loop between the tumor and the non-transformed stroma cells such as myeloid-derived suppressor cells, cancer-associated fibroblasts, and adipocytes (reviewed in [68,69]). In this manner, STAT3 can regulate a pro-carcinogenic inflammatory microenvironment and tumor immunity. For example, STAT3 activation in tumor cells can induce genes involved in the impairment of dendritic cell maturation, which results in the inhibition of T cell activation [18,70]. Conversely, blocking STAT3 activity in tumor cells induces production of the cell-extrinsic factors that enable dendritic cell maturation and function, leading to T cell activation and subsequent anti-tumor immune responses [18,70]. Recent studies have revealed STAT3 as a major player in the regulation of innate and adaptive tumor immunity and as a therapeutic target to enhance immune recognition and to break T cell tolerance of tumor cells [18,70]. Moreover, the observations that STAT3 silencing suppressed tumor cell growth more effectively in an *in vivo* microenvironment than in *in vitro* cultures strongly suggest that the role of STAT3 in facilitating tumorigenesis is mainly attributed to its effects on the tumor cell-extrinsic signaling [71,72]. 

Intriguingly, it has also been reported that high nuclear expression of Tyr705-phosphorylated STAT3 is correlated with improved survival, smaller tumors, or less aggressive histology in various tumors, including breast cancer [73,74], head and neck cancer [75], lung cancer [76], gastric cancer [77], soft tissue leiomyosarcoma [78], and advanced rectal cancer [79]. However, in pancreatic ductal adenocarcinoma, no association was seen between STAT3 phosphorylation and patient survival [80]. These studies suggest that STAT3 is not always oncogenic in cancer cells but it may behave in an opposing manner in certain tumor types. Indeed, the identity of STAT3 as an oncogene or a tumor suppressor has been questioned in different tumor types (reviewed in [30,81]).

## 4. STAT3 in Thyroid

### 4.1. STAT3 in Non-Malignant Thyroid Epithelium

STAT3 is highly expressed in normal thyroid gland. Intriguingly, STAT3 is more expressed in the right lobe than in the left lobe of thyroid, but the significance of this differential expression is currently unclear [82]. In normal thyroid glandular epithelium, Tyr705-phosphorylated STAT3 is distributed both in the cytoplasm and in the nucleus [26]. STAT3 appears to be important for thyroid function. As an effector of leptin signaling, STAT3 is involved in the regulation of neuroendocrine function of thyroid (reviewed in [83]). It has been shown that, upon stimulation of rat thyroid cells with thyroid-stimulating hormone, STAT3, but not STAT1, is rapidly phosphorylated on Tyr705 [84,85,86,87]. In this signaling, STAT3 is activated as a transcriptional activator and mediates gene expression required for the hormone-induced proliferative responses and immunomodulation [84,85,86,87].

Expression of STAT3 and its activity is altered in different thyroid diseases, showing different patterns from the expression of other STATs. For example, when surgical specimens from the patients diagnosed as having Hashimoto's disease or focal lymphocytic thyroiditis were analyzed, activated STAT1 and STAT5 were mainly detected in infiltrating cells of hematopoietic origin whereas STAT3 expression was restricted to epithelial cells [88]. This cell-type-specific expression pattern of STAT3 suggests its distinct role in growth and proliferation of regenerating thyroid follicles. Moreover, STAT3 was colocalized with the anti-apoptotic protein Bcl-2 and expression of Tyr705-phosphorylated STAT3 was associated with low levels of stromal fibrosis, suggesting that STAT3 serves as a protective factor in remodeling of the inflamed thyroid gland [88]. Of note, persisting cytokine-mediated activation of STAT3 was detected in non-lesional adjacent thyroid tissues after thyroidectomy due to different types of diseases. This STAT3 activation was associated with cytoplasmic localization of p53, which has been suggested as a risk factor for the development of neoplastic diseases [89].

### 4.2. STAT3 in Thyroid Cancer

Although aberrant STAT3 activity mediates oncogenic signaling in many different cancers, STAT3 is also known for its tumor suppressive effects (reviewed in [30]). Indeed, studies including ours have demonstrated that STAT3 can mediate tumor suppressive signal transduction in different tumor types [90,91,92,93,94]. STAT3 therefore has opposing effects on tumorigenesis, and these paradoxical STAT3 effects are also observed in certain thyroid cancer types.

#### 4.2.1. Expression of STAT3 in Thyroid Cancer

Increased expression and activation of STAT3 is detected in the tissue specimens of thyroid cancers, including lymphatic metastasis of PTC [95]. Consistent with this, a histological analysis revealed that STAT3 is distinctively expressed in PTC but not in follicular tumors, suggesting that STAT3 activation may be involved in the establishment of the papillary phenotype [96]. Moreover, a recent study detected increased nuclear-localization of Tyr705 phosphorylated STAT3 in about 57% (63 of 110) human primary PTC cases, preferentially in association with the tumor stroma [90]. On the contrary, a study has demonstrated that STAT3 activity, as determined by its affinity to DNA, is significantly lower in PTC cells than those of surrounding normal thyroid tissues [97]. In this study, tumor size larger than 2 cm was the only clinicopathologic parameter associated with lower STAT3 activity. Moreover, this study revealed an inverse-correlation between tumor size and STAT3 activity, specifically in B-Raf^V600E^-positive cases [97]. Consistent with this finding, increased STAT3 activity was inversely correlated with tumor size and the presence of distant metastases in another study [90]. However in contrast to the former study, this study detected increased STAT3 phosphorylation in multiple human primary PTC cases. These studies suggest that STAT3 expression is altered in selective types of thyroid cancer in correlation with low degree of tumor malignancy. 

#### 4.2.2. Mechanism of STAT3 Activation in Thyroid Cancer

It was originally discovered that, in the NIH3T3 model of *in vitro* tumorigenesis, the oncogene RET/PTC could induce Tyr705 phosphorylation of STAT3 [98]. In this system, STAT3 activation facilitated RET/PTC-mediated cellular transformation by regulating expression of vascular endothelial growth factor, cyclin D1, and intercellular adhesion molecule 1. This observation suggested a possibility that RET/PTC may induce STAT3 activation. Intriguingly, in the human anaplastic thyroid cancer line ARO, STAT3 could directly interact with ectopically expressed RET/PTC1 and phosphorylation of its Tyr705 did not require either JAK or c-Src kinase, indicating a possibility of direct STAT3 activation by the oncogenic kinase [98]. A recent report demonstrated that constitutive STAT3 activation can be mediated via the IL-6/gp130/JAK autocrine signaling pathway in different thyroid cancer lines that express RET/PTC, B-Raf^V600E^, or mutated Ras [90]*.* Apart from Tyr705 phosphorylation, it has also been shown that Ser727 phosphorylation can be induced by cyclin-dependent kinase 5 in response to the activation of human epidermal growth factor receptor 2 (HER2/erbB2) in the MTC cell line, TT [99]. Intriguingly, while STAT3 Tyr705 phosphorylation was not affected under this condition, overexpression of mutant STAT3 (Ser727 to Ala) dominant-negatively inhibited cyclin-dependent kinase 5-mediated TT cell proliferation, suggesting a specific role of STAT3 for HER2/cyclin-dependent kinase 5 signaling. In addition to these mechanisms, STAT3 in thyroid cancer appears to be regulated via an unexpected mechanism. In KAT-18 cells, a primary culture of anaplastic thyroid cancer, a mutant p53 that harbors a gain of mutation, *i.e*., *TP53*^G199V^ missense mutation, affected STAT3 expression status and mediated anti-apoptotic function by transcriptionally upregulating STAT3 [100]. It may also be possible that STAT3 activation in PTC is partly attributed to the mutations in receptor tyrosine kinase since receptor tyrosine kinase can activate STAT3 pathway [33] and activation mutations of epidermal growth factor receptor gene occur in PTC [101].

#### 4.2.3. Effects of STAT3 on Thyroid Cancer: Are They Tumor Suppressive?

Although STAT3 was previously reported for its effects to promote tumor cell survival and proliferation in the context of RET/PTC-tumorigenesis of NIH3T3 or TP53^G199V^ signaling in an anaplastic thyroid cancer cell line [98,100], multiple studies have also identified STAT3 as a tumor suppressor in different thyroid cancer types. For example, a recent study demonstrated that, although shRNA-mediated STAT3 knockdown did not affect *in vitro* growth of representative thyroid cancer cell lines that express high STAT3 activity, it led to the generation of larger tumors than control when STAT3-depleted tumors cells were xenografted in mice. Consistent with this result, STAT3 deficiency facilitated tumorigenesis in a murine model of B-Raf^V600E^-induced PTC [90]. To mediate tumor suppression, STAT3 increased transcription of the tumor suppresser insulin-like growth factor binding protein 7 and negatively regulated aerobic glycolysis, decreasing energy metabolism in cancer cells [90]. Therefore, STAT3 appears to function as a tumor suppressor in the background of PTC.

**Figure 1:**
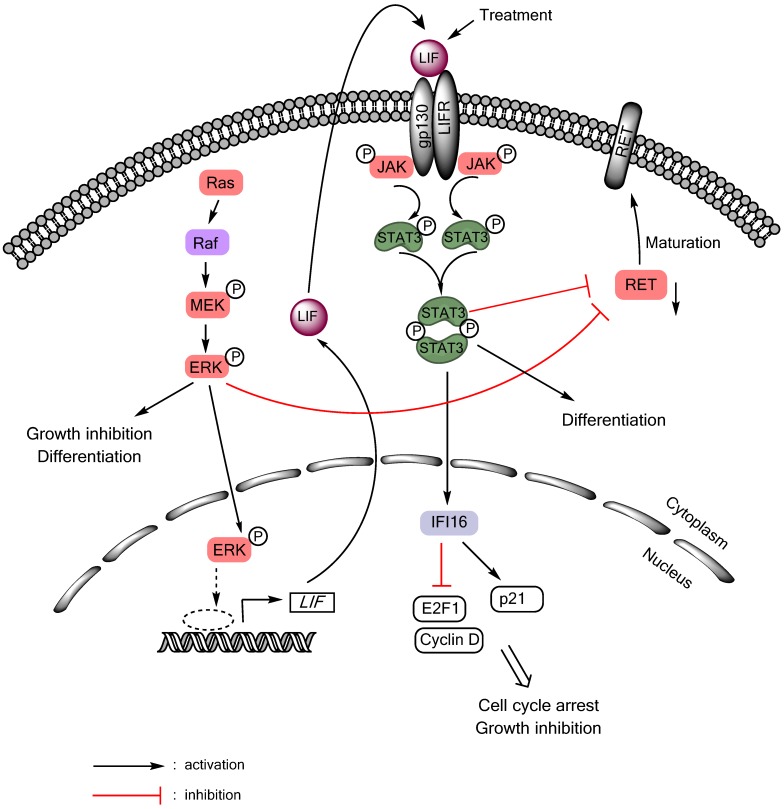
LIF/JAK/STAT3-mediated growth inhibitory signaling in medullary thyroid cancer. Activation of the Ras/Raf/MEK/ERK pathway induces RET downregulation, growth inhibition, and differentiation in MTC cells by inducing expression and secretion of LIF, which activates STAT3 in an autocrine/paracrine mode.

Autocrine/paracrine tumor suppressive signaling of the gp130/JAK/STAT3 pathway can also be activated in MTC cells in response to Ras or Raf activation (Figure 1). Although aberrant Ras or Raf activity is a central feature of many epithelial cancers, sustained activation of Ras/Raf elicits senescence-like growth arrest responses, referred to as “oncogene-induced senescence”, in primary cultured normal cells and premalignant lesions [102,103]. These phenomena are now interpreted as innate tumor-suppressive responses, which are triggered as a fail-safe anti-tumorigenic mechanism by aberrant cell proliferation signals. Intriguingly, similar responses can be induced in certain types of malignant cancer that are not transformed by Ras/Raf, including MTC [91,104,105,106,107], leading to a hypothesis that these tumor types may retain functional tumor suppressive mechanism against Ras/Raf oncogenesis. Upon Ras/Raf activation, MTC cells undergo growth arrest and differentiation within 48 hours, as manifested by cell cycle arrest in G0/G1 phases, increased calcitonin gene expression, and silenced expression of the oncogenic RET [104,106,108]. Prolonged cell culture under this condition eventually leads to expression of the senescence marker, senescence-associated β-galactosidase [109]. In different cell types, the Ras/Raf pathway mediates growth arrest by controlling key cell cycle regulatory and tumor suppressive proteins, including Rb, E2F1, cyclin-dependent kinase inhibitors, and TP53. Our previous studies indicate that, in MTC cells, this tumor suppressive signaling is connected to LIF-controlled extracellular mechanisms (Figure 1). Using biochemical analysis of the culture medium conditioned by MTC cell expressing exogenously introduced oncogenic c-Raf kinase domain, we identified LIF as being essential and sufficient to mediate the growth inhibitory signaling activated by Ras/Raf [91]. LIF mediated this growth inhibitory effect through the gp130/JAK/STAT3 pathway since anti-gp130 blocking antibody or dominant negative STAT3 blocked the effect of LIF. Subsequent analysis of STAT3-mediated gene expression programs identified IFI16 as an effector of STAT3, which regulates the S-phase transcription factor E2F1 and the cyclin-dependent kinase inhibitor p21^CIP1^ to induce cell cycle arrest in MTC cells [110]. This LIF/gp130/JAK/STAT3-mediated growth inhibitory signaling is conserved in different MTC cell lines regardless of the mutational status of *RET* [109], and could also be activated by interleukin-1β via the Raf/MEK/ERK pathway [92]. Importantly, recombinant LIF could activate STAT3 and downregulate RET and induce tumor suppression in human MTC cells xenografted in mice [111], suggesting its potential as a therapeutic reagent to treat MTC. While these studies support the tumor suppressive role of STAT3 in the context of MTC, a recent study showed that AZD1480, a small molecule inhibitor of JAK1/2, could also inhibit the growth of MTC cell lines [112]. Therefore, it may be possible that either too high or too low STAT3 activity triggers growth inhibitory responses in MTC cells. Of note, AZD1480 also showed efficacy in the STAT3-deficeint cell line, TPC-1, suggesting that the drug effect could be independent of STAT3 [112]. Together, these studies highlight the complexities of the JAK/STAT signaling in thyroid cancers.

## 5. Conclusions

Cellular context can influence the response to a signal and, indeed, STAT3 can either promote or suppress cell growth depending upon cell types. It is currently unclear how STAT3 can mediate these opposing effects. Because oncogenic stress can elicit growth inhibitory responses as an innate tumor defense mechanism, STAT3 signaling may also be networked with a similar tumor defense mechanism, which is activated in the face of aberrant STAT3 activation. It appears that this context is relevant in a subset of thyroid cancer, including PTC and MTC. Intriguingly, the effect of the Ras/Raf pathway is clearly in contrast in these two thyroid tumor types in that the pathway is constitutively active and promotes PTC cell proliferation whereas its basal activity is relatively low while its constitutive activation can induce growth inhibition in MTC cells. Nevertheless, the effect of Ras/Raf activation on STAT3 was consistent in these two tumor types in that, in both MTC and PTC, Ras/Raf activates STAT3 via the IL-6 family cytokine-mediated gp130/JAK pathway and STAT3 operates in the context of tumor suppression. It is possible that STAT3 signaling is developmental biologically programmed to be tumor suppressive in thyroid epithelium. Of note, while STAT3 played a role as a transcription factor in both thyroid cancer types, the role of STAT3 in the mitochondria was also important to mediate tumor suppressive signaling in PTC [90]. It is currently unclear whether this non-canonical STAT3 function is also important to mediate tumor suppression of MTC cells.

It is an intriguing question why the activity of Ras/Raf induces the IL-6 family cytokine-activated gp130/JAK/STAT3 pathway. Noteworthy is that an emerging issue in the study of the Ras/Raf-induced growth inhibitory signaling is the involvement of diverse extracellular soluble factors. It has been reported that cells undergoing Ras/Raf-induced senescence secrete soluble factors that can help reinforce senescence-like growth arrest responses [113,114]. Interestingly, these factors (which include insulin-like growth factor binding protein-7, plasminogen-activator inhibitor-1, and CXCR2-binding chemokines such as interleukin-8 or growth-regulated oncogene-α) were identified in a cell-specific manner. Perhaps, certain thyroid cell types may retain similar mechanisms which may be activated in response to aberrant Ras/Raf activation. Nevertheless, noteworthy is that STAT3 activity is upregulated in PTC, suggesting as-yet-unidentified benefit of STAT3 upregulation for PTC development. 
